# Blocking binding of *Bacillus thuringiensis *Cry1Aa to *Bombyx mori *cadherin receptor results in only a minor reduction of toxicity

**DOI:** 10.1186/1471-2091-9-3

**Published:** 2008-01-24

**Authors:** Taek H You, Mi K Lee, Jeremy L Jenkins, Oscar Alzate, Donald H Dean

**Affiliations:** 1 Departments of Biochemistry, Molecular Genetics and Entomology, The Ohio State University, Columbus, OH 43210, USA; 2 Department of Biological Sciences, Campbell University, Buise Creek, NC, USA; 3 Syngenta, Inc., Research Triangle Park, NC, USA; 4 Novartis Institutes for BioMedical Research, Inc., Cambridge, MA, USA; 5 Neuroproteomics Facility, Duke University, Durham, NC, USA

## Abstract

**Background:**

*Bacillus thuringiensis *Cry1Aa insecticidal protein is the most active known *B. thuringiensis *toxin against the forest insect pest *Lymantria dispar *(gypsy moth), unfortunately it is also highly toxic against the non-target insect *Bombyx mori *(silk worm).

**Results:**

Surface exposed hydrophobic residues over domains II and III were targeted for site-directed mutagenesis. Substitution of a phenylalanine residue (F328) by alanine reduced binding to the *Bombyx mori *cadherin by 23-fold, reduced biological activity against *B. mori *by 4-fold, while retaining activity against *Lymantria dispar*.

**Conclusion:**

The results identify a novel receptor-binding epitope and demonstrate that virtual elimination of binding to cadherin BR-175 does not completely remove toxicity in the case of *B. mori*.

## Background

In the final chapter of her landmark book, *Silent Spring*, Rachael Carson recommended *Bacillus thuringiensis *as a biological pesticide with less environmental impact than conventional chemical pesticides [1]. For 30 years *B. thuringiensis *formulations have been sprayed as topical pesticides with annual world-wide applications estimated at 2.3 × 10^6 ^kg [2], providing a massive experiment on its ecological and health impact. The results have demonstrated the general safety of *B. thuringiensis *for the environment and humans. The latest application method is the genetic engineering of plants to systemically express *B. thuringiensis *insecticidal proteins. This step increases specificity and generally has reduced sprayed pesticide impact on the environment in the United States and Canada.

The report of the sensitivity of monarch butterfly (*Daneaus plexippus*) to pollen of genetically modified corn [3], albeit controversial [4], and unlikely to be a threat to insects in the field [5-8] reemphasizes what has been known for almost 100 years [9], *B. thuringiensis*, despite a generally narrow host range [10], can affect certain non-target insects [11]; and the silkworm, *Bombyx mori *is a prime example [12,13].

Previous studies have indicated that surface exposed charged and hydrophobic residues in domains II loop regions of several Cry toxins are involved in receptor binding (irreversible and reversible) [14-18]. This prompted us to investigate the implications that hydrophobic residues in other areas on the surface of domain II may affect toxicity and binding. Cry1Aa was chosen because of its known structure [19]; and its high activity to both *B. mori *and the forest pest *L. dispar*. Phenylalanines at position 313, 328, 333, 335, and 461 were selected and tested for biological activity against *B. mori*, and *L. dispar*. Binding to brush border membrane vesicles (BBMV) and purified receptors of those insects were investigated.

## Methods

### Mutant construction

To construct Cry1Aa mutants, *Bacillus thuringiensis cry1Aa1 *gene (NCBI accession M11250) from pOS4101 [20], originally from pES1 [21] was subcloned into plasmid pBluescript SK(-) to generate pBN-1Aa. To accommodate the NdeI fragment containing *cry1Aa *coding region, EcoRV recognition sequence in the multiple cloning site of the vector was modified to NdeI recognition sequence by site-directed mutagenesis.

The Cry1Aa mutant toxins were constructed using single strand DNA of the wild type Cry1Aa as template. Mutagenic oligonucleotide DNA primers were synthesized from GeneMed Synthesis, Inc. (South San Francisco, CA). Single strand DNA template was prepared using Bio-Rad Mutagene mutagenesis kit and site-directed mutagenesis was performed according to manufacturer's instruction.

DNA sequencing reactions were performed in the PTC-150 Minicycler (MJ Research, Inc., MA) using the Perkin-Elmer Applied Biosystems DNA sequencing kit following manufacturer's instruction. The sequencing results were analyzed by ABI 373A DNA sequencer.

### Protein purification

The crystal inclusion bodies of the Cry1Aa and its mutant toxins were expressed and purified from *E. coli *MV1190 containing pBN-1Aa as described [22]. The purified crystal proteins were solubilized in 50 mM Na_2_CO_3 _containing 10 mM DTT, pH 9.6 at 37°C with shaking at 220 rpm for 5 h. The solubilized protoxin was digested with 2% (w/w) trypsin (Sigma) at 37°C for 3 h, at a trypsin/protoxin ratio of 1:20 by mass. When obvious incomplete digestion was observed after visualizing on SDS-10 % polyacrylamide gels, the protoxins were further digested with an additional dose of 1 % (w/w) trypsin for 2 h. Toxins were further purified as the monomeric fraction on a Hiload 16/60 Superdex 200 Prep Grade column (GE Healthcare) in either HBS (HEPES-buffered saline, 10 mM Hepes, pH 7.4, 150 mM NaCl, 3.4 mM EDTA) for Biacore analysis, or 20 mM phosphate buffer, pH 7.4 with an Äkta Explorer FPLC (GE Healthcare). To ensure toxin existed as a monomer in solution, dynamic light scattering for molecular size detection was employed using a DynaPro 801 (Protein Solutions, Inc.). Concentration of protoxins and toxins was estimated by Coomassie Protein Assay Reagent (Pierce) and the purity was examined on the SDS-10 % PAGE [23].

### Toxicity bioassays

*L. dispar *eggs were kindly provided by Gary Bernon (U.S. Department of Agriculture, Otis Methods Development Center, Beltsville, MD). Eggs were hatched and reared on artificial diet (Bio-Serv, Frenchtown, NJ). *B. mori *eggs were kindly provided by Ross Milne (Canadian Forestry Service, Sault Ste. Marie, Ontario, Canada). Eggs were hatched and reared on mulberry (*Morus albus*) leaves from trees received from the Canadian Forestry Service, Sault Ste. Marie, Ontario, Canada) and grown in the College of Biological Sciences Entomology Greenhouse. Insects were reared at ambient temperature and humidity. Activities of toxins were determined with 2–3 day old *L. dispar *and *B. mori *larvae by the surface contamination method as described [24] and [20], respectively, using trypsin activated, column purified monomeric toxin. Toxins were diluted in 50 mM sodium carbonate buffer (pH 9.5), and 50 μg samples were applied per well (2 cm^2^) on artificial diet in 24-well tissue culture plates. Two larvae were placed in each well and the mortality was recorded after 5 days. Bioassays were repeated at least 4 times. The effective dose estimates (LC_50_, 50% lethal concentration of toxin) were calculated using PROBIT analysis [25].

### BBMV binding assays

Mutant toxins were tested for binding to brush border membrane vesicle (BBMV) of *B. mori*. The BBMVs were prepared from midguts of the last instar larvae of *B. mori*, by the magnesium precipitation method as described [26]. Twenty μg of toxin was iodinated with 1 mCi of Na^125^I (Amersham) in 50 mM sodium carbonate buffer (pH 9.5) in the presence of one IODO-BEAD (Pierce). Incubation was for 15 min at room temperature. Labeled toxins were separated from the unlabeled free iodine using the Excellulose GF-5 column (Pierce). The specific activity of the labeled Cry1Aa was 0.75 μCi/μg protein. Heterologous competition binding assays were performed as described previously [22]. Ten μg of BBMV (amounts of BBMV refers to BBMV protein throughout) was incubated with 2 nM of ^125^I-labeled toxins in 100 μl of 8 mM NaHPO_4_, 2 mM KH_2_PO_4_, 150 mM NaCl, pH 7.4, containing 0.1 % BSA, for 1 h at room temperature in the presence of increasing amounts of corresponding unlabeled competitors (from 0 to 500 nM). The bound toxins were separated from unbound toxins by centrifugation at 15,500 rpm (22,000 × g) for 10 min. The pellet containing the bound toxins was washed twice with binding buffer, and the radioactivity in the resulting pellet was counted in a gamma counter (Beckman).

### Receptor purification

Purified *B. mori *aminopeptidase N and cadherin-like protein were obtained from *B. mori *midguts that were dissected from 4^th ^or 5^th ^instar larvae. BBMV were prepared by the general method of Wolfersberger [27] as described [22]. *B. mori *BBMV (10 mg in 10 ml) was solubilized in 5 mg/ml CHAPS zwitterionic detergent (Roche) overnight at 4°C with gentle rocking. Solubilized BBMV was centrifuged at 10,000 × g for 10 min and supernatant was concentrated to 2 ml by Amicon YM30 ultrafiltration. The sample was loaded on a Q Sepharose HR 10/30 anion-exchange column with an ÄKTA Explorer (GE Healthcare) after column activation. A salt gradient (0–1 M NaCl was used to elute BBMV proteins. The low salt buffer (buffer A) consisted of 20 mM Tris, 5 mM MgCl, 0.4 mg/ml CHAPS, pH 8.6, and the high salt buffer used was buffer A + 1 M NaCl. All fractions were tested for APN activity by the LpNA assay. Briefly, 390 μl of sample are mixed with 10 μl of 2 mM leucine-p-nitroanalide (containing a leucine-phenylalanine dipeptide). A yellow chromophoric change indicates aminopeptidase N activity, defined as the ability to cleave a neutral amino acid from the N-terminus of a polypeptide. Cry1Aa binding ability was also checked by slot blotting fractions to PVDF membrane and probing with biotinylated Cry1Aa [28]. Biotinylation was done with a Biotin Labeling Kit (Roche). Fractions with Cry1Aa-binding ability and APN activity were concentrated and loaded on a HiLoad 16/60 Superdex 200 Prep Grade column (GE Healthcare) (120 ml bed volume). Fractions eluting around 120 kDa, the MW of APN, were collected, concentrated by Amicon (Millipore) ultrafiltration on YM30 membranes, and protease inhibitor was added before freezing. Several kinds of APNs occur in the midgut of *B. mori *[29]. Our preparation may contain more than one of these. Anion-exchange fractions with Cry1Aa-binding ability but without APN activity were also size-purifed, and fractions eluting around 175–250 kDa, the MW of BtR175, were collected and stored. Additionally, the purified fractions were loaded on a 6% SDS-PAGE gel, then transferred overnight at 40 V to PVDF. The membrane was probed with biotin-Cry1Aa and biotin-Cry1Aa F328A. Cry1Aa bound to a single band at 120 kDa in the APN fraction and a single band at 201 kDa in the cadherin fraction. Cry1Aa F328A bound only to a single 120 kDa band in the APN fraction but bound no observable bands in the cadherin fraction.

### Surface plasmon resonance with purified midgut receptors

*B. mori *APN and *B. mori *cadherin were immobilized (100–200 ng for each) on three flowcells of a single CM5 sensor chip by the amine-coupling method (Biacore AB). Receptors were diluted into ammonium acetate, pH 4.2 prior to immobilization. An HBS (pH 7.4) buffer (discribed above under Protein Purification) flow rate of 50 μl/min was used for all injections. Randomized toxin concentrations varying from 100 nM to 1000 nM were injected (110 μl) over the receptor surfaces. Surfaces were regenerated with 6 μl pulses of 10 mM NaOH, 250 μM ethylene glycol, pH 11.0 at 100 μl/min. Flowcell 4 (blank) responses were subtracted from all response curves and data were fitted using BIAevaluation 3.0. The curves were fit to a simple 1:1 Langmuir binding model to obtain apparent rate constants (A + B ↔ AB).

## Results and discussion

**Figure 1:**
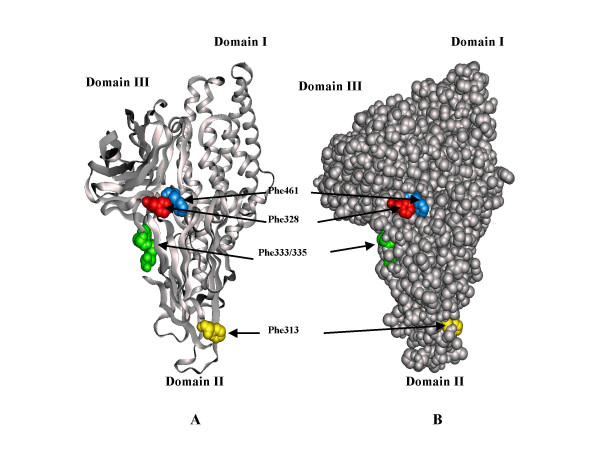
**Structure of Cry1Aa insecticidal protein showing surface phenylalanine residues (red) which were changed to alanine**. A, ribbon diagram model to illustrate the secondary structure of domains I (alpha helical), II (beta strands) and III (beta sandwich). B, space filling model, to illustrate the surface exposure of the phenylalanine residues (red). Molecular models were generated using QUANTA (Molecular Simulations, Inc.)

To investigate the role of surface hydrophobic residues on receptor binding, the mutant proteins, F313A, F328A, and F461A; and, the double mutant protein, F333A-F335A, were expressed at similar levels to that of wild type Cry1Aa (35–50% total cell protein [20]) and produced stable toxin peptides by trypsin activation. Stability refers here to the trypsin resistant state of the 65 kDa toxin. Another alanine mutant toxin, F338A, was expressed at a low level or was degraded during trypsin activation (no further studies were conducted with this mutant). The positions of these residues on the surface of the Cry1Aa toxin structure are shown (Fig. 1).

Toxicity of wild type Cry1Aa and mutant toxins against *L. dispar*, and *B. mori *larvae are given in Table 1 as LC_50 _values of each toxin. Against *L. dispar*, none of the mutant proteins show significant differences in toxicity. To *B. mori*, mutant toxin F328A displayed four-fold reduced toxicity, while F313A, the double mutant F333A-F335A, and F461A displayed overlapping confidence limits with the wild type Cry1Aa toxin.

**Table 1:** Biological activities of Cry1Aa and mutant toxins to *L. dispar*, and *B. mori*. Toxicity is measured as LC_50 _in ng/ccm.

Toxin	LC_50 _against *L. dispar*	LC_50 _against *B. mori*
Cry1Aa (Wt)	1.59 (0.76–2.64)	10.45 (7.14–14.12)
F313A	1.68 (0.86–2.72)	17.34 (11.70–21.74)
F328A	1.80 (0.98–2.80)	41.52 (24.69–66.76)
FF333,335AA	2.18 (1.30–3.26)	15.47 (8.8–25.5)
F461A	1.73 (1.18–2.46)	17.1 (12.7–22.7)

**Figure 2:**
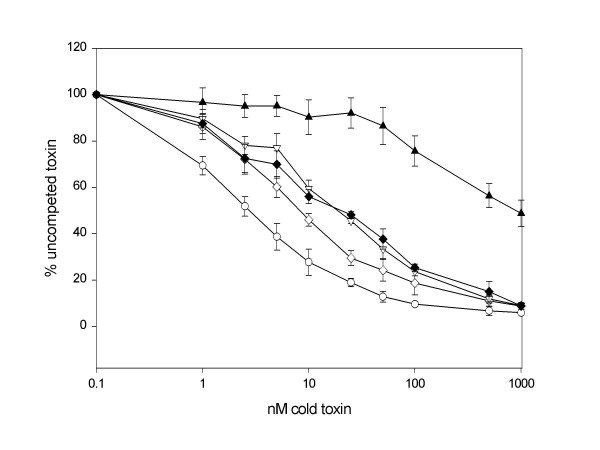
**Competition with labeled Cry1Aa and mutant toxins to *B. mori *brush border membrane vesicles (BBMV) (bars represent error of n = 4)**. Cry1Aa wild type protein, (○-○); F313A mutant protein, (◊-◊); F333A-F335A, mutant protein (▽-▽); F461A mutant protein, (◆-◆); and, F328A mutant protein, (▲-▲). % uncompeted toxin indicates the % of labeled toxin remaining bound to BBMV in the presence of unlabeled cold toxin competitor.

To investigate the mechanism by which mutant protein F328A reduces toxicity to *B. mori*, we examined toxin-binding properties to BBMV. BBMV competition binding assays of wild type Cry1Aa and mutant toxins demonstrated that F328A exhibited little competition with the wild type toxin until the highest amounts of competing toxins. This reduced affinity is consistent with its reduced toxicity. Mutant proteins F313A, F461A, and the double mutant, F333A-F335A, toxins displayed slightly decreased binding affinity compared to the wild type Cry1Aa (Fig. 2).

We further examined binding properties of these mutant proteins to the purified known *B. mori *Cry1Aa binding proteins from the *B. mori *midgut. Previous studies demonstrated the purification of Cry1Aa binding proteins (receptors), aminopeptidase N (APN) and cadherin-like receptor, from *B. mori *[12,13]. We have previously examined binding of Cry1Aa, Cry1Ab, Cry1Ac and hybrid domain-substitution mutants involving Cry1Aa and Cry1Ac by the surface plasmon resonance with purified *B. mori *binding proteins [30]. We found that Cry1Aa binds to *B. mori *cadherin-like proteins with a Kd of 2.6 nM and to *B. mori *APN with a Kd of 75 nM. Binding of Cry1Ab and Cry1Ac was negligible to either one of these binding proteins. Domain substitutions indicated that domain III from Cry1Ac did not affect binding, indicating that either the binding epitopes of domain III from Cry1Aa and Cry1Ac are the same or that they play little role in binding the cadherin-like proteins.

**Figure 3:**
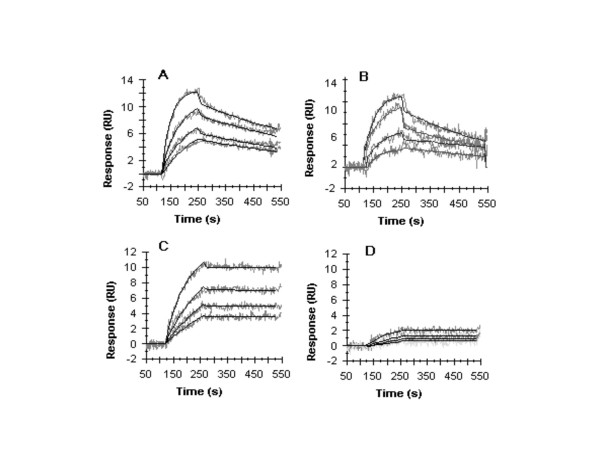
**Binding of Cry1Aa and the mutant toxin F328A to the APN and cadherin-like protein purified from *B. mori *measured by surface plasmon resonance (SPR) using a Biacore 1000**. Representative BIAcore response curves for toxin injections at 200 (lower curve), 300 (second curve from bottom), 500 (third curve from bottom), and 1000 nM (top curve). Experimental curves (grey) are shown overlaid with fitted curves (black) obtained with the 1:1 Langmuir binding model. The same APN-bound chip is used in figures A and B, and the cadherin-bound chip is used in figures C and D. (A) Cry1Aa wt binding to *B. mori *APN. (B) F328A binding to *B. mori *APN. (C) Cry1Aa wt binding to *B. mori *cadherin-like protein. (D) F328A binding to *B. mori *cadherin-like protein.

In this study, we purified both of these binding proteins from *B. mori *midgut tissue and analyzed binding properties of wild type and mutant proteins using surface plasmon resonance on a BIAcore 2000 (Fig. 3). The wild type Cry1Aa binding to cadherin-like receptor was ka = 1.3 × 10^4 ^M^-1^s^-1 ^(+/- 6.1 × 10^3^), kd = 3.3 × 10^-5 ^s ^-1 ^(+/- 1x10^-5^), K_D_= 2.6 nM; and to *B. mori *APN were 2.1 × 10^4 ^M^-1^s^-1 ^(+/- 1.3 × 10^2^), kd = 1.5 × 10^-3^s^-1 ^(+/- 1 × 10^-5^), K_D _= 75 nM. Mutant protein F328A showed significantly lower binding to cadherin-like receptor but bound slightly less (2-fold lower) to APN than wild type. Its binding to *B. mori *APN was 1.8 × 10^4 ^M^-1^s^-1 ^(+/- 2.9 × 10^2^), kd = 2.3 × 10^-3 ^s^-1^, and K_D _= 132 mM; while to *B. mori *cadherin, the F328A mutant toxin yielded apparent rate constant values to cadnerin: ka = 1.5 × 10 ^3 ^M^-1^s^-1 ^(+/- 9.0 × 10 ^3^), kd = 9.1 × 10^-5 ^s^-1 ^(+/- 8 × 10^-9^), K_D _= 60.7 nM, a 23-fold lower binding affinity than wild type Cry1Aa. This receptor binding data suggested that the phenylalanine residue at 328 might play an important role in binding to the cadherin-like receptor, which led to the reduction in toxicity. Other mutant toxins, F313A, F333A-F335A, and F461A did not show any changes in binding to either APN or cadherin-like receptor(data not shown).

The cadherin-like protein has been reported to be a functional Cry1Ab receptor [31-34], while APN was reported as a functional receptor for Cry1Ac [35-37] and Cry1C [38]. More recently an additional receptor, BR-270, has been reported as an anionic brush border membrane glycoconjugate that show tight binding to a number of Cry toxins [39]. The role of this receptor in the overall mechanism of action of Cry toxins is unknown and its response to mutant toxins is unreported. It is possible that only one of these is a functional receptor, leading to lethality, while the others serve as non-functional toxin-binding molecules. Or, perhaps a more complicated, multifaceted mechanism is involved, as proposed recently [40].

One unexpected finding in these experiments was that F461A, F313A and F333A-F335A mutant toxins did not change the binding properties to either APN or cadherin-like proteins (not shown), although these mutant proteins did affect overall binding to BBMV. This may be due to the possibility that these mutations might affect binding to a binding protein other than APN and the cadherin-like protein. Taking into account the fact that F313 is located on a different face from F333 and F335 as indicated in the Fig 1, it is suggested that these phenylalanine residues might be involved in binding to different receptors or different binding steps.

After the work on this paper was completed we became aware of a paper [41] reporting a *B. thuringiensis *strain, AF101, which exhibited lower activity toward *B. mori*. The authors reported that an altered Cry1Ab toxin, responsible for the lower activity, differed in three residues and the carboxy-terminal portion from wild type Cry1Ab. The three residues were S261A (a partially buried residue), S560N and F328L, the critical residue found in our work with the different Bt toxin, Cry1Aa. Since Cry1Ab does not bind significantly to APN or cadherin [30], one might speculate that F328 may also interact with other receptors.

Our observation that mutation in F328 to A affected binding to *B. mori *cadherin but not to *L. dispar *cadherin indicates that different toxin-receptor interactions take place in different insects. This principle was noted earlier in comparing the action of mutants of Cry1Ab and their action on *Manduca sexta *and *Heliothis virescens *[24].

Our understanding of certain aspects of the function of the *B. thuringiensis *insecticidal proteins, especially in the area of receptor binding has also been enhanced by protein engineering techniques [10,42-44]. Briefly, the previously described receptor-binding epitopes, best understood in Cry1Ac, are in the loops at the bottom of domain II, and N-acetylgalactosamine binding pocket in domain III [43-45].

Protein engineering techniques have allowed improvements of proteins as well as understanding of their function [46]. Improvements in toxicity against target pests, as much as 36-fold, have been reported for *B. thuringiensis *toxins [47-49]. In the present paper we demonstrate the proof of principle that site-specific mutagenesis can render these biopesticides more specific by reducing activity to a non-target insect.

## Conclusion

In the present paper, we have observed a new receptor-binding epitope around residue 328 in domain II, between the previously observed epitopes. Mutations in this region specifically reduced toxicity 4-fold to *B. mori *by blocking binding to *B. mori *cadherin, BTR175, whereas binding to *B. mori *APN was unaffected. The results demonstrate that virtual elimination of binding to cadherin BR-175 does not completely remove toxicity in the case of *B. mori*.

Through a combination of learning more about the mechanism of action of these biopesticides, rational application of protein engineering techniques and serendipity, better insecticidal proteins can be developed that have improved activity against target pests and reduced activity against certain non-target insects. The overall aim in this effort is to protect agricultural crops and reduce non-specific pesticides to achieve increased food production while protecting the environment.

## Authors' contributions

THY contributed to the conception of the study, conducted most of the molecular genetic studies, MKL contributed to the conception of the study and helped with the molecular genetic studies. JLJ conducted the surface plasmon resonance binding studies. OA   repeated all of the bioassays helped revise the manuscript. DHD conceived of the study, participated in its design and coordination, and corrected, refined and polished the manuscript. All authors read and approved the final manuscript.
